# Altering brain dynamics with transcranial random noise stimulation

**DOI:** 10.1038/s41598-019-40335-w

**Published:** 2019-03-11

**Authors:** Onno van der Groen, Jason B. Mattingley, Nicole Wenderoth

**Affiliations:** 1 Neural Control of Movement Laboratory, Health Sciences and Technology, ETH Zurich Zurich, 8057 Switzerland; 2 School of Medical and Health Sciences, Edith Cowan University, Joondalup, Western Australia 6027 Australia; 3 Queensland Brain Institute, The University of Queensland, St Lucia, Queensland 4072 Australia; 4 School of Psychology, The University of Queensland, St Lucia, Queensland 4072 Australia; 5 Canadian Institute for Advanced Research (CIFAR), Toronto, Canada

## Abstract

Random noise can enhance the detectability of weak signals in nonlinear systems, a phenomenon known as stochastic resonance (SR). This concept is not only applicable to single threshold systems but can also be applied to dynamical systems with multiple attractor states, such as observed during the phenomenon of binocular rivalry. Binocular rivalry can be characterized by marginally stable attractor states between which the brain switches in a spontaneous, stochastic manner. Here we used a computational model to predict the effect of noise on perceptual dominance durations. Subsequently we compared the model prediction to a series of experiments where we measured binocular rivalry dynamics when noise (zero-mean Gaussian random noise) was added either to the visual stimulus (Exp. 1) or directly to the visual cortex (Exp. 2) by applying transcranial Random Noise Stimulation (tRNS 1 mA, 100–640 Hz zero -mean Gaussian random noise). We found that adding noise significantly reduced the mixed percept duration (Exp. 1 and Exp. 2). Our results are the first to demonstrate that both central and peripheral noise can influence state-switching dynamics of binocular rivalry under specific conditions (e.g. low visual contrast stimuli), in line with a SR-mechanism.

## Introduction

Noise is detrimental for the transfer of information in linear systems^1^. However, in nonlinear systems such as the brain, noise can enhance information transfer via a stochastic resonance (SR) mechanism^1,2^. SR can be experimentally observed when noise is added to a non-linear system which enhances (i) the output of the system e.g. by improving the signal-to-noise ratio (SNR)^3,4^, (ii) the signal amplitude^5^ or (iii) the degree of coherence within neural networks^6^. In humans, the SR-effect has been observed in multiple sensory modalities when both signal and experimentally controlled noise are added to the peripheral nervous system^7–10^. Recently, we have demonstrated that central mechanisms of perception are also sensitive to an SR-effect^3^. We showed that transcranial random noise stimulation (tRNS), a type of non-invasive brain stimulation, applied over visual cortex can enhance the detection performance of weak subthreshold visual stimuli.

Theoretical considerations predict that SR not only plays a role in signal enhancement but that it can also influence the dynamics of bistable-systems^11^. One paradigm that allows the observation and measurement of how the brain dynamically transitions between different perceptual states is binocular rivalry. Binocular rivalry is a perceptual phenomenon that occurs when different stimuli are simultaneously presented to each eye. During binocular rivalry visual awareness switches spontaneously between the two stimuli^12^ so that at any given time participants perceive either one of the two images (exclusive percept) or a combination of both (mixed percept).

Models of binocular rivalry propose that it reflects a competition between neural populations coding for each image^13^. The neural population coding for the dominant percept inhibits neurons that code for the suppressed image. However, over time the inhibition of the dominant population becomes weaker due to adaptation or fatigue, which allows the suppressed image to become dominant^13,14^. This results in a deterministic anti-phase oscillation of the firing rates of the two neuronal populations^15^. If adaptation was the only driving factor of binocular rivalry, however, perception would change fairly regularly. In fact, the dynamics of binocular rivalry are highly nonlinear and stochastic, leading to the proposal that noise associated with the activity of the two neuronal populations causes a random distribution of dominance durations^16^.

Noise is thought to play a crucial role in the occurrence of the perceptual switches, and it has been suggested to represent an essential driving force of rivalry dynamics^17,18^. Consequently, there are many successful models of binocular rivalry which contain a noise component to describe the rivalry dynamics^15,19,20^. While it has been demonstrated that rivalry dynamics can be influenced by adaptation^21^, the effect of adding noise directly to the brain during binocular rivalry has not been empirically tested. Rivalry dynamics can conceptually be represented by a double-well energy landscape, with each well representing one of the images. Neuronal adaptation can be represented as changes in the energy-landscape: adaptation reduces the depth of the well so that the current state is less stable, thus making a switch to the competing percept more likely. Adding noise to such a system can change its dynamics in a specific way^11,22^. Here we investigated whether the attractor dynamics of binocular rivalry can be modulated by adding noise to either the visual stimulus or directly to the visual cortex with tRNS^23,24^.

Previously it has been demonstrated that 10 minutes of continuous tRNS increases cortical excitability for up to an hour after stimulation^24^. In the current study, however, we were interested in the direct online effects of noise on rivalry dynamics. Therefore, noise and no-noise conditions were randomly intermixed, and noise was applied non-continuously to reduce the likelihood of overall changes in neural excitability. Two experiments were performed: In Experiment 1, noise was added to low contrast or high contrast visual stimuli to test whether an SR-effect is induced when noise is added to the periphery. In Experiment 2, we added noise to the visual cortex with tRNS to test whether central mechanisms of perception are sensitive to an SR-effect. The results of these experiments suggest that rivalry dynamics can be influenced by noise when there are three stable states, namely, perception of the left-eye image, the right-eye image and a combination of both images. Since SR occurs preferentially for weak but not for strong stimuli^3,7–10,25^ we tested  a low- and a high-contrast condition in both experiments. In order to make clear predictions as to which outcome parameters were most likely to be affected by adding noise to rivalry dynamics, we simulated different experimental conditions with a computational model^19^ prior to data collection.

## Results

### Computational modelling results

**Figure 1:**
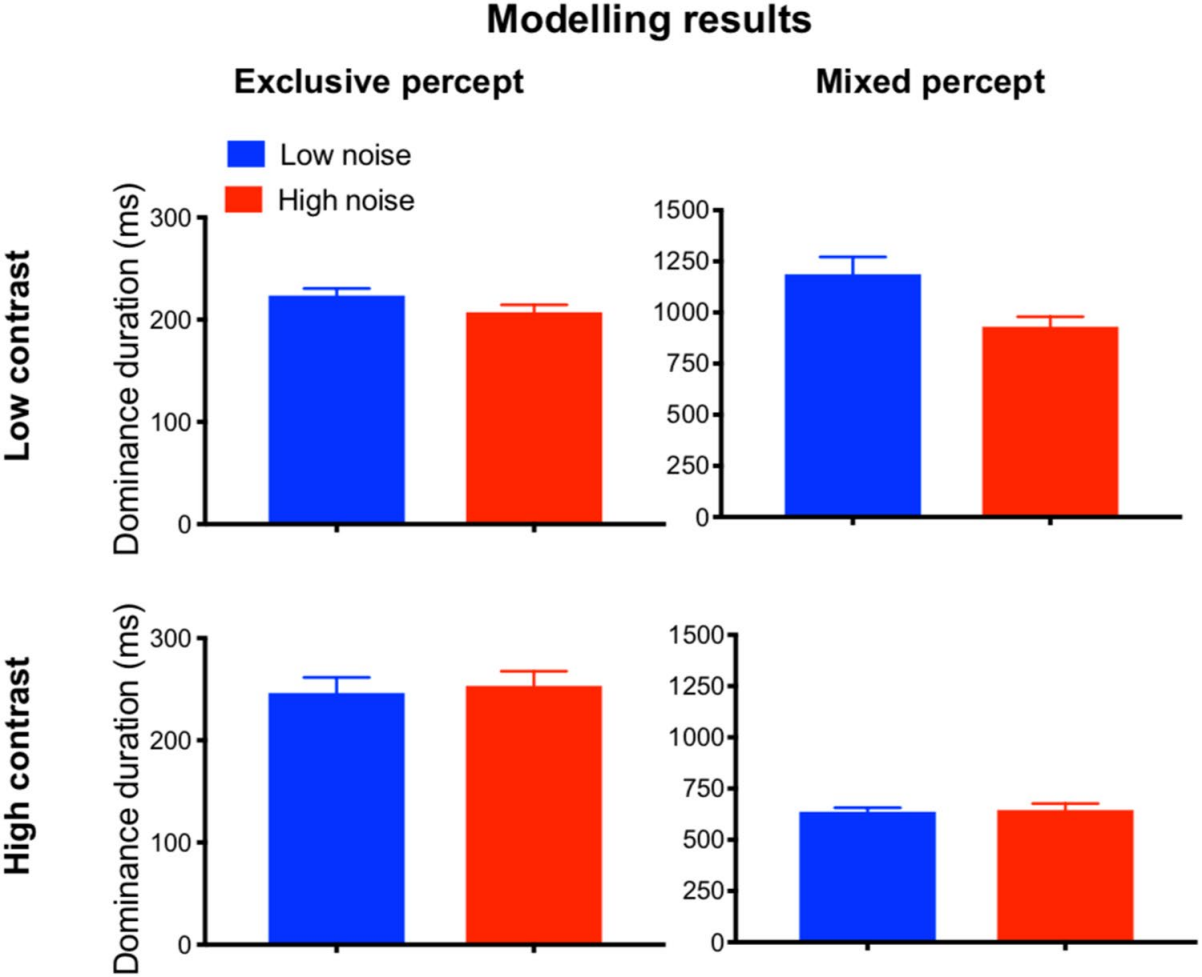
Computational modelling results. We modelled the effect of adding noise to a rivalry model, and determined dominance durations for the exclusive and mixed percepts. The modelled results indicate that adding noise to the rivalry process reduces the duration of the mixed percept for low contrast visual stimuli by 22%. For the high contrast visual stimuli this reduction was 1%. For the exclusive percept, the dominance duration decreased with an increasing noise level for the low-contrast visual stimuli (7% reduction) and increased for the high-contrast visual stimuli (3% increase). Error bars represent standard deviations (s.d.).

We used a conventional binocular rivalry model^19^ which relies on competition between neurons tuned to orthogonal orientations. Perceptual switches result from the interplay of mutual inhibition between these neurons and a noise component. We added a common noise term to the neuronal populations since tRNS is most likely to add correlated noise to many neuronal populations. When adding noise to the model, the duration of exclusive percepts decreased by 7% for the low contrast stimulus (low noise mean[s.d.]: 223.5[7.1] ms, high noise mean[s.d.]: 207.4[7.2]) ms) and increased by 3% for the high contrast stimulus (low noise mean[s.d.]: 246.3[15.5] ms, high noise mean[s.d.]: 253.3[14.4] ms, Fig. 1, left panel). The strongest effect of adding noise was observed as a substantial reduction of the mixed percept (Fig. 1, right panel) which was, however, different depending on contrast intensity: there was a stronger reduction of mixed percept durations for low contrast trials (−22% change, low noise mean[s.d.]: 1187.3[84] ms, high noise mean[s.d.]: 931[48.2]) ms) than for high contrast trials (1% change, low noise mean[s.d.]: 636.8[19.9] ms, high noise mean[s.d.]: 645.3[31.9]) ms). These modelling results are in line with previous research suggesting that mixed percept duration is affected by neuronal noise^26–28^. Based on these studies and our own modelling findings, the primary outcome measure for the subsequent binocular rivalry experiments was the mixed percept duration. In particular, we hypothesized that the mixed percept duration would be more strongly influenced by adding noise to low contrast stimuli than to high contrast stimuli. As a secondary outcome measure we further calculated the number of perceptual switches over the 12 minutes of modelled rivalry. The number of switches increased most with increased noise in the low-contrast condition (low noise mean[s.d.]: 310.3[11.41], high noise mean[s.d.]: 397.8[14.4]; 28% change), whereas there was no change in the number of switches in the high-contrast condition (low noise mean[s.d.]: 425.3[16.6], high noise mean[s.d.]: 423.8[12.6]; 0% change).

### Behavioural results

#### Experiment 1

**Figure 2:**
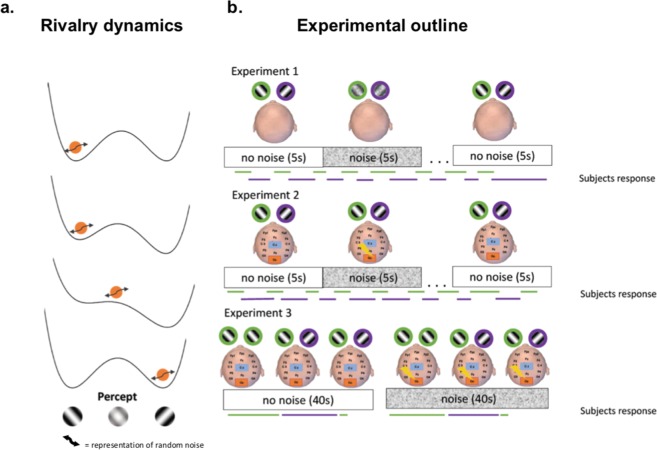
(**a**) Representation of rivalry dynamics. Binocular rivalry can be represented by a double-well energy landscape. The orange ball determines the current percept. Binocular rivalry is thought to be driven by adaptation and noise. Adaptation changes the landscape, meaning one of the wells becomes less shallow. Noise (arrow) causes the percept to change more quickly when the boundary between the two wells is low. (**b**) Experimental outline. In Experiment 1 noise (zero-mean Gaussian random noise) was applied to the visual stimulus for 5 seconds followed by 5.5–7 seconds of no stimulation. The same noise was applied to the left and right eye. The noise intensity was subthreshold for each individual participant. Experiment 2 followed the same protocol, except that the noise was applied to the visual cortex directly with tRNS (zero-mean Gaussian random noise, 100–640 Hz).

Participants were seated in front of the monitor and viewed the images foveally through a mirror stereoscope (Fig. 2). The task for the participants was to continuously report on a keyboard whether they perceived the left or right tilted grating, or a mixture of both.

**Figure 3:**
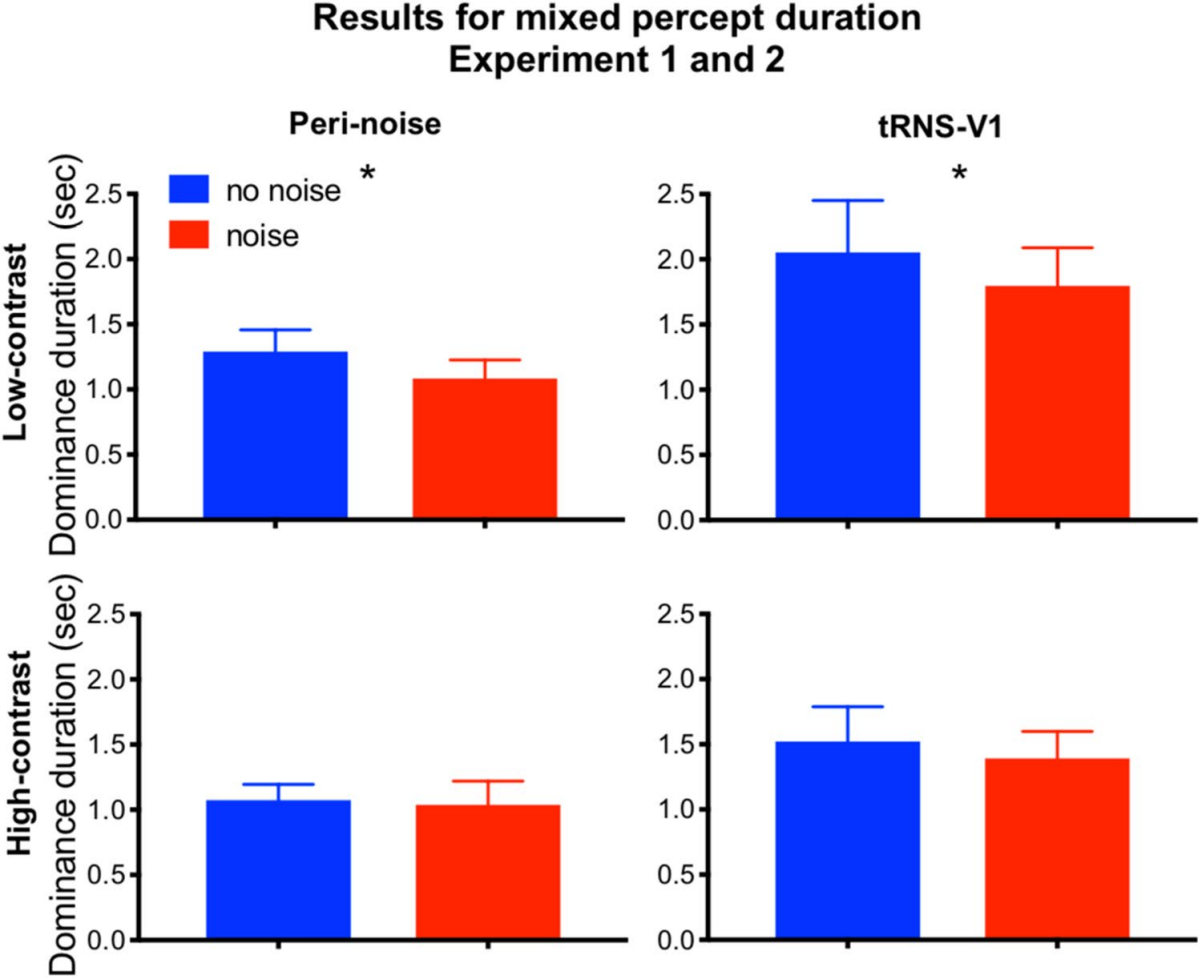
Behavioural results from Experiments 1 (left) and 2 (right). Adding noise to the visual stimulus significantly reduced the dominance duration of the mixed percept for low contrast visual stimuli by 16%, whereas the dominance duration for the high contrast visual stimuli was reduced by 4%. In Experiment 2 (right) the noise was added to the cortex with tRNS. This resulted in a reduction of 15% in median mixed percept duration. The dominance duration for the high contrast visual stimuli was reduced with 6% There was no significant effect of adding noise on the dominance duration of the exclusive percept. Error bars represent standard errors (s.e.m.).

We investigated two separate cohorts (each n = 10) which performed the task either with low contrast or high contrast stimuli (see methods for further details). Adding noise to low contrast visual stimuli during binocular rivalry significantly reduced the median dominance duration of the mixed percept (low-noise mean[s.d.]: 1.29[0.53]sec, high-noise mean[s.d.]: 1.08[0.45] sec; t(9) = 2.938, p = 0.017; Cohen’s d = 0.65; −16% change in dominance duration; Fig. 3). Adding noise to high contrast visual stimuli did not significantly affect the mixed dominance duration (low-noise mean[s.d.]: 1.07[0.38] sec, high-noise mean[s.d.]: 1.04[0.58] sec; t(9) = 0.356, p = 0.730; Cohen’s d = 0.08; −4% change in dominance duration). Note that we observed a medium effect size for the low contrast stimuli, whereas there was only a small effect for high contrast stimuli suggesting that the noise effect is larger for the former.

Additionally, we tested the number of perceptual switches as a secondary outcome parameter: The number of perceptual switches was not significantly affected by peripheral noise for low contrast stimuli (low noise mean[s.d.]: 173.2[41.8], high noise mean[s.d.]: 181.7 [29.1]; t(9) = −1.284, p = 0.23; 5% change) or high contrast stimuli (low noise mean[s.d.]: 259.2[119.4], high noise mean[s.d.]: 258[113.9]; t(9) = 0.272, p = 0.79; 0% change).

#### Experiment 2

In Experiment 2 we added noise directly to the visual cortex with tRNS. Consistent with previous investigations, no participant reported awareness of the tRNS stimulation during de-briefing^29,30^. Adding noise to visual cortex with tRNS yielded a similar pattern of results to Experiment 1. Specifically, the median mixed dominance duration of the low contrast visual stimulus was significantly reduced (low-noise mean[s.d.]: 2.05[1.53] sec, high-noise mean[s.d.]: 1.79[1.14] sec; t(14) = 2.263, p = 0.04; Cohen’s d = 0.41, −13% change in dominance duration). Adding noise to a high contrast visual stimulus did not significantly affect the mixed dominance duration (low-noise mean[s.d.]: 1.51[1.03] sec, high-noise mean[s.d.]: 1.39[0.8] sec; t(14) = 0.930 p = 0.368, Cohen’s d = 0.16, −8% change in dominance duration). The number of perceptual switches was not significantly affected by peripheral noise for low contrast stimuli (low noise mean[s.d.]: 186.5[71.6], high noise mean[s.d.]: 194.2[61]; t(14) = −1.581, p = 0.136; 4% change) or high contrast stimuli (low noise mean[s.d.]: 184.7[49.9], high noise mean[s.d.]: 183.3[50.4]; t(14) = 0.428, p = 0.68; 0% change).

## Discussion and Conclusion

Our results demonstrate for the first time that adding noise to a visual stimulus during binocular rivalry can significantly influence rivalry dynamics by reducing mixed percept durations. The same results were obtained when noise was added to the visual cortex directly with tRNS. This effect only occurred when the visual stimuli had a low contrast. These results are in line with the predictions made by the binocular rivalry model (see Fig. 1). Exclusive percept dominance durations in Experiments 1 and 2 were not affected by noise.

Adding noise to the visual stimuli (peripheral noise) resulted in the same behavioural results as adding noise to the brain directly with tRNS (central noise), namely, a significant reduction in mixed percept duration when the stimuli had a low contrast. No effect was observed when the stimuli were presented with a high contrast, suggesting that the stimulus contrast is a crucial parameter. Previously we have shown that noise added either peripherally or centrally can enhance performance on a contrast detection task^3^ for weak visual stimuli according to an SR-effect. An important difference between these two noise application methods is the location in the nervous system at which they influence visual processing for the first time. The initial influence of peripheral noise is at the receptor level. tRNS, on the other hand, influences visual processing at the cortical level. By applying tRNS during binocular rivalry we found that cortical noise can causally influence mixed percept duration, as predicted by the model.

Our secondary outcome measurement, the number of perceptual switches, changed in line with the model prediction but the effect was small and did not reach significance in the behavioural experiment. Note that we adopted an existing model which was developed and validated in a different experimental context^19^. Since the model was not optimized for the current experiment, its predictions were qualitatively similar to our behavioural results but not quantitatively. For example, the absolute dominance durations predicted by the model were much shorter than those observed experimentally. This discrepancy might have arisen because we did not model inter-subject variability, which has been shown to be substantial in response to transcranial electrical stimulation^31^. It is unclear how the intensity of “neural noise” induced by tRNS over visual cortex could be determined at the level of the individual, or how it could be parametrized in the model. More sophisticated, biologically plausible models might shed further light on these questions.

The processes underlying the occurrences of mixed percepts during binocular rivalry are unclear, but one possible mechanism involves changes in the strength of mutual inhibition between the two neuronal pools, each coding for a different percept^32,33^. It is thought that stronger stimuli (stimuli with a higher signal-to-noise ratio) result in an increase in mutual inhibition, which leads to a reduction in the mixed percept duration^33^. Adding noise could have strengthened mutual inhibition due to an increase in the SNR of the stimulus representation in the brain. Related to this finding, a recent study demonstrated that alcohol intake enhances mixed percept duration^34^. It has been shown in cats that alcohol reduces the signal-to-noise ratio (i.e. increases noise) in primary visual cortex^35^. In our study, we likely increased the SNR in visual cortex, resulting in a reduction of mixed percept duration. Another possible mechanism that effects mixed percept duration is a change in the balance between excitatory and inhibitory neural activity^36^. An imbalance between cortical excitation and inhibition is an important factor in autism spectrum disorder (ASD) models^28^. However, it is still unknown whether lower biological noise levels are also involved in ASD^37^. Interestingly, our finding that only mixed percepts are sensitive to a noise effect is supported by a study of binocular rivalry in ASD^28^. Robertson and colleagues did not find any difference between healthy controls and people with ASD on exclusive percept duration, but did find an increase in mixed percept duration in people with ASD^28^.

Our rivalry stimuli were relatively large (4°), which is larger than the receptive fields of individual V1 cells. Therefore, participants could have experienced ‘piecemeal rivalry’, which occurs when patches of both images are perceived together^38^. We did not test how much piecemeal rivalry participants experienced, but previous research suggests that it would have been less prominent in the low contrast condition than in the high contrast condition^18^. Previous research suggests that piecemeal rivalry is not affected by peripheral noise^39^.

It is known that tRNS is able to enhance cortical excitability after 4 minutes of continuous stimulation^40^, which results in a change in the excitation-inhibition balance. Here we alternated tRNS and non-tRNS runs in random order across participants, making it unlikely that the observed effects were driven by an overall change of excitability. Additionally, tRNS runs in Experiment 2 applied the stimulation for relatively short periods of 5 seconds, and these were followed by 5.0 to 7.7 s with no stimulation to reduce effects on overall excitability. Besides this, adding noise to the visual stimulus resulted in similar effects, and as far as we know cortical excitability cannot be modulated by peripheral noise. Therefore, our results are most likely explained by an SR-effect that modulates perceived stimulus contrast. It has been previously demonstrated that binocular rivalry is modulated by an SR-mechanism when evoked by peripheral noise^41^. In contrast to our study, Kim and colleagues did not add noise to the rivalry process, but changed the strength of the driving signal by changing the contrast of the two images in counter phase. The idea is that SR will occur when the driving signal matches biological noise levels. The reason we found no effect of noise on the dominance duration of exclusive percepts in our study could because the noise added might not have been optimal to introduce an SR-effect in a bistable system. The noise properties we applied were based on the findings of our previous study^3^ where we demonstrated that tRNS and peripheral noise are able to enhance performance on a contrast detection task. The fact that we might not have added an optimal noise level for everyone might explain why the effect sizes for the tRNS noise were relatively low.

In order to determine whether the tRNS-induced electrical noise reached the cortex, we have previously estimated that the applied field strength is too small to directly depolarize single neurons^3^. However, it has been proposed that the tRNS effect is amplified because thousands of neurons are modulated simultaneously^42^. Thereby even small depolarization may be enough to elicit action potentials (APs) in neurons that are close to their firing threshold, which results in a weak stimulus reaching the threshold for AP generation earlier.

Although it is still debated where in the brain binocular rivalry is resolved, we targeted the visual cortex. In particular, we selected an electrode montage and chose an intensity which is suitable for stimulating visual cortex as indicated by previous modelling work^3^. The reason we targeted this region is because neuronal activity in primary visual cortex (V1) is linked to subjective percepts during binocular rivalry^43–45^. Changes in higher order brain areas, including frontoparietal cortex, are thought to reflect the consequences of perceptual alternations, rather than being the cause of them^46^. However, there is also evidence that frontoparietal activity might play a causal role^47–49^. Evidence that mixed percepts may be more influenced by low-level visual processes rather than higher order processes^50,51^ supports the interpretation that our effect is driven by stimulation of these low-level visual cortices. The results of our study show that the effect of noise on the rivalry dynamics occurs at a cortical level. It has been shown before that peripheral noise can enhance the perceived contrast of a visual stimulus at the receptor level^10,52^. Therefore, finding an effect of adding noise peripherally does not necessarily mean that rivalry dynamics are sensitive to an SR effect, because any such effect could be explained at the receptor level.

In conclusion, our results are the first to demonstrate that mixed percept durations can be altered by applying noise to the visual cortex directly with tRNS, likely due to an enhancement of stimulus contrast. Our results open up new ways of manipulating noise levels within the brain, and provide a better understanding of the role noise plays in the brain when it is in a dynamical state.

## Materials and Methods

The study was approved by the Kantonale Ethikkomission Zürich, Switzerland (KEK-ZH-Nr. 2014-0269) and was conducted in accordance with the Declaration of Helsinki. Informed consent was obtained from all participants before the start of the experiment.

### Computational model of rivalry dynamics

We applied a computational model to predict how noise influences rivalry dynamics^19^. We used a conventional binocular rivalry model which relies on competition between neurons tuned to orthogonal orientations. The model relies on mutual inhibition and it also includes a noise component. The model contains two different neuronal populations and calculates the difference in firing rate between these populations. Over time inhibition on the suppressed neuronal population weakens, which eventually results in the inhibited population becoming dominant and suppressing the other population. When one population is firing fully the other population will be inhibited, resulting in the percept related to the fully firing population being dominant. When the difference in firing rate between the two populations is small then there is no winning population, resulting in a mixed percept. We introduced a criterion for mixed percepts, which was a difference in firing rate between the two neuronal populations of smaller than 0.1. In calculating dominance durations, we only included dominance times longer than 150 ms. In the model, we changed the strength of the visual stimulus and the amount of noise according to our experimental parameters. All other model parameters were identical to the original model parameters^19^. The simulation was run 100 times in order to derive an estimate of the variability of the model.

### General procedures of Experiments 1 and 2

All experiments took place in a dark and quiet room. Visual stimuli (left and right tilted gratings) were generated using MATLAB version 2012b (MathWorks, Natick, USA) and the Psychophysics Toolbox^53–55^. Stimuli were controlled by a HP Elitedesk 800 G1 running Windows 7 (2009). Stimuli were presented on a Sony CPD-G420 colour monitor with a calibrated linearized output at a resolution of 1280 × 1024 pixels, with a refresh rate of 75 Hz. The two images were placed on the horizontal meridian to the left and right hand side of the screen on a uniform grey background (53 Cd/m^2^). The visual stimuli were orthogonal Gaussian gratings (orientation ± 45°, diameter 3 cm, contrast 20%, visual angle 4°, spatial-frequency 2.2 cycles per degree) surrounded by a white square (diameter 3.5 cm), to promote binocular fusion. Participants were seated in front of the monitor and viewed the images through a mirror stereoscope from a distance of 45 cm while resting their head on a chin rest. The images were viewed foveally (through the stereoscope).

Each participant performed 8 runs of binocular rivalry, each lasting 3 minutes. The task for the participants was to continuously report on a keyboard whether they perceived the left-eye or right-eye tilted grating or a mixture of both. In each experiment the noise was applied in 4 runs to either the screen (Exp. 1) or directly to the visual cortex with tRNS (Exp. 2). The order of the noise runs was randomized over participants. In these runs the noise was applied for 5 seconds followed by a 5.5–7 seconds’ interval of no stimulation, and in total 18 times per run. In each experiment participants received a total of 360 seconds’ visual noise (exp. 1) or tRNS (exp. 2). Participants performed one practice run without any noise before the start of the experiment.

### Experiment 1: Peri-noise condition

In this experiment, we tested whether adding noise (zero-mean Gaussian random noise) to the visual stimuli influences binocular rivalry dynamics. The same noise was applied to the left and right eye. Previous research demonstrated that a noise intensity corresponding to 60% of threshold effectively induces an SR-effect^3^. Therefore, we used a simple up-down method to estimate each individual’s 60% correct noise-threshold before the experiment started, where 75% correct reflects threshold. We tested two cohorts of participants, one with a low contrast visual stimulus (20% contrast, n = 10, mean age = 23 years) and the other with a high contrast visual stimulus (70% contrast, n = 10, mean age = 24 years).

### Experiment 2: tRNS-V1 condition

In this experiment, we tested the hypothesis that adding noise directly to the visual cortex with tRNS can influence rivalry dynamics. Noise was applied centrally with tRNS (100–640 Hz, zero-mean Gaussian random noise). Electrode placement was determined using the 10–20 system. The stimulation electrode was placed over the occipital region (Oz in the 10–20 EEG system) and the reference electrode over the vertex (Cz in the 10–20 EEG system). Electroconductive gel was applied to the contact side of the electrode (5 × 7 cm) to reduce skin impedance. Electrodes were held in place with a bandage. This setup has been demonstrated to be suitable for stimulation of the visual cortex^3,56,57^. More specifically, previous work has estimated the electrical field strength produced by this montage in visual cortex^3^. In brief, modelling the electrical field for specific frequencies between 100 and 500 Hz (50 Hz steps,) and the current flow across the brain revealed sufficiently focal stimulation of visual cortex. The induced electrical field was estimated to reach a maximum of 2 V/m with all frequencies being transmitted to the brain. Stimulation was delivered by a battery-driven electrical stimulator (DC-Stimulator Plus, neuroConn). An intensity of 1 mA was applied since it has been demonstrated that this intensity effectively induces an SR-effect in most subjects^3^. The maximum current density in this experiment was 28.57 μA/cm^2^, which is within current safety limits^58^. We again tested two cohorts of participants, one where the visual stimulus had a low contrast (20% contrast, n = 15, mean age = 23 years) and the other where the visual stimulus had a high contrast (70% contrast, n = 15, mean age = 24 years). We tested more participants in Experiment 2 because it was expected there would be less power to detect a reliable effect of tRNS relative to peripheral visual noise^3^.

### Data analysis and statistics

Statistical analyses were performed using SPSS (version 20.0, IBM). The same statistical procedures were applied to both experiments. For each participant, we calculated the median dominance duration for the exclusive percepts. We also calculated the mixed percept dominance durations. Times where no button was pressed, or when the dominance duration was shorter than 150 ms, were excluded from analysis. Dominance durations terminated by the end of a block were not included in the analyses. In each experiment, we also calculated the number of perceptual switches. To test for the effect of the added noise, dominance durations and number of perceptual switches were subjected to a two-sided within-subject t-test. The α-level was set to 0.05 for all tests.

## Data Availability

The datasets generated during and/or analysed during the current study are available from the corresponding author on reasonable request.
